# Depression-Related Brain Connectivity Analyzed by EEG Event-Related Phase Synchrony Measure

**DOI:** 10.3389/fnhum.2016.00477

**Published:** 2016-09-26

**Authors:** Yuezhi Li, Cheng Kang, Xingda Qu, Yunfei Zhou, Wuyi Wang, Yong Hu

**Affiliations:** ^1^Laboratory of Neural Engineering, Shenzhen UniversityShenzhen, China; ^2^Shenzhen Kangning HospitalShenzhen, China; ^3^Department of Psychological and Brain Sciences, University of CaliforniaSanta Barbara, Santa Barbara, CA, USA; ^4^Department of Orthopaedics and Traumatology, The University of Hong KongPokfulam, Hong Kong

**Keywords:** depression, electroencephalogram, oddball paradigm, event-related phase coherence, connectivity

## Abstract

This study is to examine changes of functional connectivity in patients with depressive disorder using synchronous brain activity. Event-related potentials (ERPs) were acquired during a visual oddball task in 14 patients with depressive disorder and 19 healthy controls. Electroencephalogram (EEG) recordings were analyzed using event-related phase coherence (ERPCOH) to obtain the functional network. Alteration of the phase synchronization index (PSI) of the functional network was investigated. Patients with depression showed a decreased number of significant electrode pairs in delta phase synchronization, and an increased number of significant electrode pairs in theta, alpha and beta phase synchronization, compared with controls. Patients with depression showed lower target-dependent PSI increment in the frontal-parietal/temporal/occipital electrode pairs in delta-phase synchronization than healthy participants. However, patients with depression showed higher target-dependent PSI increments in theta band in the prefrontal/frontal and frontal-temporal electrode pairs, higher PSI increments in alpha band in the prefrontal pairs and higher increments of beta PSI in the central and right frontal-parietal pairs than controls. It implied that the decrease in delta PSI activity in major depression may indicate impairment of the connection between the frontal and parietal/temporal/occipital regions. The increase in theta, alpha and beta PSI in the frontal/prefrontal sites might reflect the compensatory mechanism to maintain normal cognitive performance. These findings may provide a foundation for a new approach to evaluate the effectiveness of therapeutic strategies for depression.

## Introduction

There are many treatment modalities and available pharmacotherapies for depressive disorders. It would significantly benefit the clinicians in terms of selecting the most appropriate treatment if there is a clear and sensitive measurement to show the efficiency during treatment initiation.

Major depression is believed to alter the brain activity in thalamocortical and corticocortical circuits. Measurement of event-related potentials (ERPs) has been widely used to investigate the cognitive process in patients with depressive disorders. In particular, major depression leads to a decrease in P300 amplitude in response to target stimuli in oddball tasks. The P3 decrement in depressed patients suggested a deficit in temporoparietal regions involved in context updating and memory processing (Bruder et al., 2012). The reduction of P300 to targets in major depression may also reflect abnormality of circuit pathway between the frontal and parietal areas (Kemp et al., 2010). The majority of reported studies have focused on the amplitude and latency measures of P300. Functional connectivity, defined as the temporal correlation between spatially remote neurophysiological events, is considered an important mechanism for the coordination of activity between different cell assemblies (Fingelkurts et al., 2005; Goldenberg and Galvan, 2015). Alterations in brain functional connectivity under resting-state condition contributed to disorganization syndrome in depression (Fingelkurts et al., 2007; Leuchter et al., 2012; Fingelkurts and Fingelkurts, 2015). Further studies are required to explore differences in functional connectivity between patients with depression and controls in cognitive tasks.

Coherence is a well-established indicator of functional connectivity, and has been successfully used to examine the spatial integration at short and long distances in the brain (Klimesch et al., 2008; Del Percio et al., 2011; Fries, 2015). Event-related coherence, which can be interpreted as event-related phase coherence (ERPCOH) or event-related linear coherence, is a frequency-domain measure of synchronization that is used to analyze electroencephalogram (EEG) data between two channels or component activities in sets of trials (Delorme and Makeig, 2004). The ERPCOH ensures that only the relative phases of the two spectral estimates at each trial are taken into account. Previous studies seldom assessed ERPCOH during the visual oddball paradigm, or attempted to determine phase coherence or compared changes between healthy controls and pathological participants, such as patients with depression (Guntekin et al., 2008; Kukleta et al., 2009). Because there were a few resting-state EEG studies that revealed increased functional connectivity in patients with depression (Fingelkurts et al., 2007; Leuchter et al., 2012), we proposed a hypotheses of functional connectivity disorganization during attentional processes in major depression, and we expected that the disorganization of brain connectivity in major depression provides a new interpretation and assessment of P300 alterations during the target detection process.

To test the brain connectivity of patients with depression during the visual P300 oddball paradigm with 64-channel EEG signals, we used the ERPCOH, as defined by the phase synchronization index (PSI) values. PSI uses values between 0 and 1 (Mizuhara et al., 2005; Mizuhara and Yamaguchi, 2007). In particular, the PSI value of each electrode pair was computed in both the target and nontarget conditions to define significant pairs. Then, the PSI value of significant pairs in patients with depressive disorder and healthy controls was compared in the four frequency bands. Finally, the brain connectivity network based on PSI value was established to identify the depression-related alterations in connectivity.

## Materials and Methods

### Participants

A total of 33 subjects were divided into healthy control and patient groups. Nineteen healthy subjects were recruited from the hospital or university staff, while 14 patients with major depressive disorders were recruited from the Depression and Anxiety Disorders Clinic at Shenzhen Kangning Hospital. All enrollments were examined by two senior psychiatrists with experience of more than 10 years by using the Structured Clinical Interview for DSM-IV Axis I Disorders, Clinician Version (SCID-CV; First et al., 1996). In the control group, all subjects passed the examination of SCID-CV and had no history of depression. In the patient group, subjects with anxiety disorder, a history of head trauma, or who had taken drugs in the 4 weeks before the study were excluded, there were eight unmedicated first episode patients, four relapsing patients with minimum drug-free period of 5 months and two patients with minimum drug-free period of 4 weeks. The characteristics of the participants are shown in Table 1. This study was approved by the local institutional ethics review board. The study was conducted in line with the Code of Ethics of the World Medical Association (Declaration of Helsinki). A written informed consent was taken from each subject before the experiment. A consecutive series of outpatients in the Depression and Anxiety Disorders Clinic in 1 month were tested based on inclusion criterion.

**Table 1:** **Characteristics of participants**.

	Healthy controls (*n* = 19)	Depressive disorder alone (*n* = 14)
**Gender**
Male	11	8
Female	8	6
**Age (years)**
Mean	29.3	31.2
SD	7.0	7.8
**Education level (years)**
Mean	14.2	13.0
SD	2.3	2.7
**HRSD 17**
Mean	1.6	23.9
SD	1.3	3.8
**HARS**
Mean	4.2	11.9
SD	1.7	4.6

*The severity of depression and anxiety was quantified by using HRSD 17 and HARS. HRSD 17 = Hamilton Rating Scale for Depression (17 items); HARS = Hamilton Anxiety Rating Scale. There were no significant differences in age or education level across groups (P > 0.05), and all groups had equivalent gender distribution*.

### Experimental Procedure

A classical visual oddball task was used in the experiments (Yantis et al., 2002; Bledowski et al., 2004). The stimuli were solid blue shapes presented in a random series, presented once every 2 s, and each stimulus lasted for 75 ms. Participants were instructed to press a button as quickly as possible to respond to the target stimulus. The paradigm included two task types (square task and circle task; Table 2), and both tasks included three blocks with 120 stimuli each. The whole experiment session lasted ~24 min. The task order was counterbalanced across participants. Prior to the experiment, a practice block (10 target and 20 standard stimuli) was presented to familiarize the participants with the paradigm.

**Table 2:** **Stimulus characteristics**.

Stimulus	Rate of occurrence	Task type	
		Square task (viewing angle)	Circle task (viewing angle)
Target	10%	1.38°▪	1.21°•
Nontarget	90%	1.53°▪	1.36°•

*Rate of occurrence, shape and visual angle of stimuli for the visual oddball tasks. In the experiment, stimuli were presented in blue. The shape sizes are relative and presented for illustrative purposes*.

### EEG Recording

Sixty-four channel EEG signals were recorded by a NeuroScan system (NeuroScan Lab, Charlotte, NC, USA) with a standard EEG cap based on the extended 10–20 system of electrode positions. The EEG signals were amplified with band-pass filtered at 0.05–100 Hz and sampling rate of 1000 Hz. EEG data referenced to the nose were recalculated off-line against the average reference. VEOG and HEOG were recorded with two pairs of electrodes, one placed above and below right eye, and the other 10 mm from the lateral canthi. Electrode impedance was maintained below 5 kΩ throughout the experiment. EOG artifacts were corrected using automated ocular artifact reduction module in Scan (NeuroScan Lab). The EEG was digitally filtered with a low-pass filter at 30 Hz (24 dB/Octave), segmented in epochs of 1200 ms, time-locked to stimulus onset and included a 200 ms pre-stimulus baseline. For each segment, a baseline correction for the data 200 ms prior the stimulus was performed. Then, trials contaminated by amplifier clipping, bursts of EMG activity or peak-to-peak deflection exceeding ±120 μV were excluded from averaging.

The ERPs were computed separately for the target and nontarget conditions. The individual difference waveforms were calculated by subtracting the ERP to nontarget stimuli from that to target stimuli. Grand average difference waves were obtained from the average of all participants. The individual difference waveforms were also filtered into delta, theta, alpha and beta frequency bands respectively, and then averaged across the participants to obtain the grand average difference waveform in the four frequency bands.

The largest positive peak between 300 and 600 ms was identified as P300. Latencies and amplitudes of P300 were submitted to a three-way repeated-measure ANOVA with electrode sites (F3/4/z, C3/4/z, P3/4/z; those electrode sites were used to represent left frontal, midline frontal, right frontal, left central, midline central, right central, left parietal, midline parietal, right parietal scalp regions) and hemisphere (left, midline, right) as within-subject factors and group (Healthy group, depression group) as the between-subjects factor. Greenhouse-Geisser correction was used, and corrected *p*-values were reported. All data were presented with means ± standard error.

### EEG Data Analysis

In considering the effect of volume conduction and reference signals on the phase synchronization measurements (Sun et al., 2012), recorded EEG trials were transformed to the scalp current density at each electrode site by applying the spherical Laplace operator to the voltage distribution on the scalp with Brain Vision Analyzer software (Brain Products, Germany). This procedure was performed with the order of the splines = 4, the maximum degree of the Legendre polynomial = 10, and a precision of 10^−5^. Next, we down-sampled the scalp current density to 500 Hz and exported it into EEGLAB, running under the MATLAB environment (Mathworks, Natick, MA, USA) for further analysis. The signal of the scalp current density was convoluted by complex Morlet’s wavelet w(*f,t*):

(1) $$\text{w}(f,t)\;=\;(\frac{1}{\sqrt{\pi \delta_{t}}}\exp(-t^{2}/2\delta_{t}^{2})\exp(i2\pi ft)$$

where w(*f,t*) is the product of a sinusoidal wave at frequency *f*, with a Gaussian function with a standard deviation δ_t_ proportional to the inverse of *f* (Delorme and Makeig, 2004). In EEGLAB, the number of wavelet cycles is set as 0.5 and the lowest frequency time window as 0.5 s which made the lowest frequency analyzed be 1 Hz and the number of cycles in the wavelets used for higher frequencies continue to expand slowly, reaching half (0.5) the number of cycles in the equivalent window at its highest frequency (20 Hz)^1^.

The time-frequency transform of the scalp current density at frequency *f* and time *t* at electrode *a* was computed using the equation:

(2) $$F_{k}^{a}(f,t)\;=\;S_{k}^{a}(t)⊗w(f,t)$$

where $S_{k}^{a}$ is the signal of scalp current density at electrode *a* of trial *k.* We then defined the PSI between electrodes *a* and *b* with the following equation:

(3) $${\text{PSI}}_{a,b}(f,t)\;=\;\frac{1}{n}\sum_{k\;=\;1}^{n}\frac{F_{k}^{a}(f,t)F_{k}^{b}{\left(f,t\right)}^{*}}{\left|F_{k}^{a}(f,t)F_{k}^{b}(f,t)\right|}$$

where *n* is the number of available trials. PSI_a,b_(*f,t*) was computed by 1 Hz steps from 1 Hz to 20 Hz. The set of PSI_a,b_(*f,t*) is termed PSI below.

To identify the task-dependent modulation of the PSI, we compared the PSIs of the individual participants between the target and nontarget conditions with a two-sample *t*-test. To estimate the confidence interval for the *t*-value, we used a bootstrap procedure (Mizuhara et al., 2005; Parks et al., 2016), which frees us from making unverifiable assumptions about the data (e.g., probability distribution). Using the 2000 bootstrapped re-samples that were obtained from PSI_a,b_(*f,t*) in the target and nontarget conditions, the *t*-values were computed with the two-sampled *t*-test for each individual participant. Based on the bootstrapped *t*-values, the one-sample *t*-test was used to generate a distribution that allowed us to determine the threshold of the *t*-value. We counted the number of electrode pairs for which the *t*-value exceeded the threshold (*p* < 0.05) for each frequency. Next, we computed the sum of the *t*-value of these electrode pairs for each individual participant. The sum of *t*-values was then compared between the depressive and healthy groups to determine the significance of difference for each frequency. The frequency with maximal significant difference in each frequency band was selected for further clustering analysis.

### EEG Phase Synchronization Clustering

We classified the phase synchronization pairs with the following steps. First, we computed the correlation coefficient of the PSI time series of every significant electrode pair on an intra-participant basis. These correlation coefficients were tested for significance with a one-sample *t*-test on an inter-participant basis (one-tailed *p* < 0.05 or *p* < 0.01). Based on the correlation coefficient of the PSI, electrode pairs with significant phase synchronization indices were categorized into a set of clusters. If the synchronization indices between two electrode pairs had a significantly high cross-correlation coefficient, the two were categorized into elements of the same cluster. Next, in an obtained cluster, every electrode pair had at least one other electrode pair with a high correlation of PSI. Electrode pairs in one cluster did not have high correlations of PSI with electrode pairs in any other cluster. The threshold for the correlation coefficients and the number of electrode pairs determined the number of clusters automatically. In addition to the test of the correlation coefficient, the threshold of the number of the cluster size was used to reduce the risk of a type I error (cluster size ≥12 pairs; Mizuhara and Yamaguchi, 2007).

### Cross-Correlation Analysis Among Phase Synchronization Indices

We used the time series of PSIs at the frequency of interest in the target condition to compute the cross-correlation coefficient. The cross-correlation was computed for every two time series of PSIs with the following equation:

(4) $$r(\tau )\;=\;\frac{\frac{1}{N}\sum_{i\;=\;1}^{N}\left(f_{i}-\bar{f}\right)\times (g_{i+\tau }-\bar{g})}{\sqrt{\frac{1}{N}\sum_{i\;=\;1}^{N}{\left(f_{i}-\bar{f}\right)}^{2}\times \sum_{i\;=\;1}^{N}{\left(g_{i}-\bar{g}\right)}^{2}}}$$

where *r(τ)* is the cross-correlation coefficient *r* at the time lag *τ* between two time series of indices (*f_i_* and *g_i_*), *N* is number of time points in the time series, and $\bar{f}$ and $\bar{g}$ are the mean values of the time series *f_i_* and *g_i_*, respectively. To compute the average cross-correlation of the indices between the electrode pairs within each cluster, all cross-correlations computed over time series of PSIs in the cluster were averaged on an intra-participant basis, and the averaged cross-correlations were averaged across participants. To compute the average cross-correlation of the indices between two clusters, all cross-correlations computed over the time series of PSI respectively in one cluster and in another cluster were averaged on an intra-participant basis, and the averaged cross-correlations were averaged across participants.

## Results

### Behavioral Data

The behavioral results are summarized in Table 3. Reaction time did not differ significantly across groups. Similarly, the hit rate for target stimuli did not differ significantly across groups. The false-positive rate for the nontarget stimulus also did not differ significantly across groups (*p* > 0.05).

**Table 3:** **Mean reaction time and performance rates of the two groups in visual oddball paradigm**.

	Healthy control	Depression
Reaction time (ms)	589.7	593.5
Target hit rate	82.9%	81.8%
False positive rate (nontarget )	0.98%	1.95%

### ERP Results

**Figure 1:**
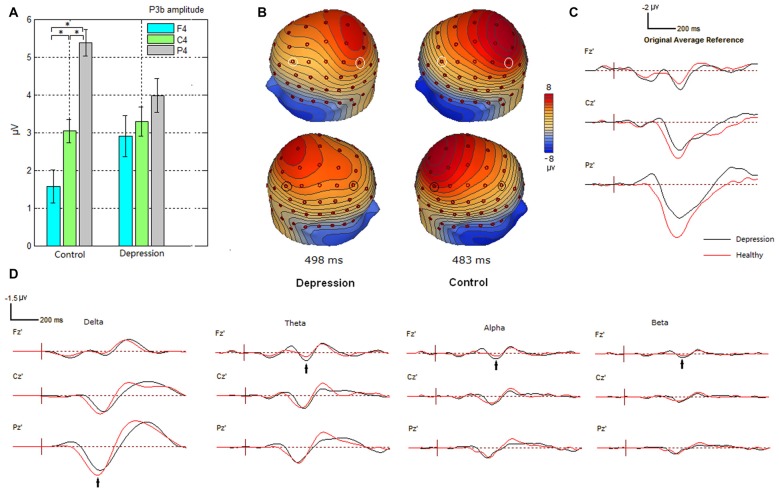
**Alteration of P300 amplitudes at frontal, central and parietal sites in depression group. (A)** Bar graph to show the effect of electrode site on P300 amplitude in the right hemisphere in participant groups. Amplitude was plotted as mean ± standard error. **(B)** Altered scalp voltage maps of the P300 in the right hemisphere in the depressive patients. Peak latencies of P300 are respectively indicated. The circled electrode sites are F3/4 and P3/4. **(C)** Grand average difference event-related potentials (ERPs) in healthy and depressive groups. **(D)** Grand average difference ERPs in different frequency bands in healthy and depressive groups. *indicates significant difference (*P* < 0.05).

For P300 analysis, analysis of variance showed significant differences between the two-way interaction of group × electrode site (*F*_(2,62)_ = 3.239; *p* = 0.023) and the three-way interaction of group × electrode site × hemisphere (*F*_(4,124)_ = 2.308, *p* = 0.025). Further analysis showed that the interaction of group × electrode site was more conspicuous in the right hemisphere (*p* = 0.05). *Post hoc* analysis revealed that for the depressive group, there was no significant effect of electrode site (*p* > 0.05). For healthy control, the P300 amplitudes were significantly larger at parietal sites than at frontal sites (*p* < 0.001; see Figure 1A). Figure 1B shows increased frontal P300 and decreased parietal P300 in the right hemisphere in the patients with depressive disorder group. The grand average difference ERP waves of P300 are shown for healthy participants and patients with depressive disorder in Figure 1C. Moreover, grand average difference ERP waves in delta, theta, alpha and beta frequency bands are shown in Figure 1D. The main decrease in P300 at parietal sites in the ERP wave of patients with depressive disorder was found in the delta band, while the main increase in P300 at frontal sites in the ERP wave of patients with depressive disorder was found in the other three frequency bands.

### Task-Dependent EEG Phase Synchronization

**Figure 2:**
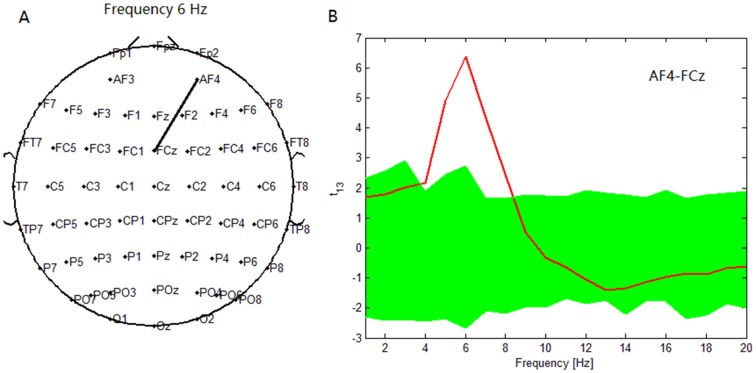
**In depressive patients, (A) maximum significance of phase synchronization index (PSI) increase during target condition relative to that during nontarget condition was found between AF4 electrode and FCz electrode at 6 Hz. (B)** *t* statistical values for the difference of the PSI between target condition and nontarget conditions for the pair (AF4-FCz) across subjects. At this pair, the PSI in the 4–7.5 Hz theta band was higher during target condition with a peak at 6 Hz. Green band represents the 95% confidence interval constructed using the bootstrap method.

To compare the PSI in the target condition with that during the nontarget condition from 1 to 20 Hz, we counted the pairs of electrodes that showed significant PSI modulations between the nontarget and target conditions (*p* < 0.05). Note that the statistical threshold for the PSI was uncorrected for the multiple comparisons about the electrode pairs, although we used the bootstrap method to correct for the high correlation of the time and frequency fields caused by the wavelet analysis (Mizuhara and Yamaguchi, 2007). As a result, in patients with depressive disorder, the maximum significance of the increase in PSI during the target condition relative to the nontarget condition was found between the AF4 and FCz electrodes at 6 Hz (Figure 2).

**Figure 3:**
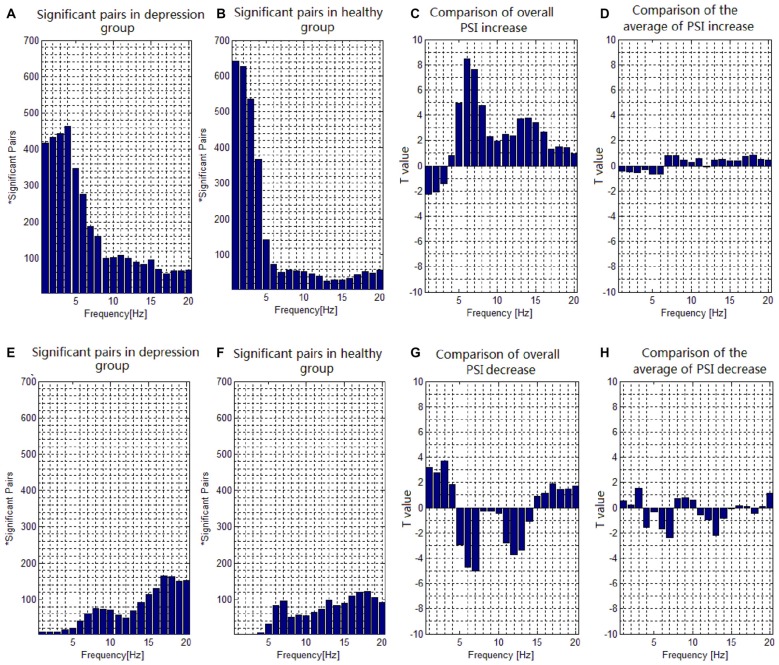
**Task-dependent increase and decrease of the electroencephalogram (EEG) phase synchronization indices across all subjects. (A)** The number of electrode pairs exhibiting greater indices during the target condition than that during nontarget condition in depression group. **(B)** The number of electrode pairs exhibiting greater indices during the target condition than that during nontarget condition in healthy controls. **(C)** Comparison of the overall increase of PSI among all significant pairs in depressive patients with that in healthy subjects with a two-sample *t*-test. **(D)** Comparison of the average value of PSI increase among all significant pairs in depressive patients with that in healthy subjects with a two-sample *t*-test. **(E)** The number of electrode pairs exhibiting lower indices during the target task than that during nontarget condition in depression group. **(F)** The number of electrode pairs exhibiting lower indices during the target task than that during nontarget condition in healthy control. **(G)** Comparison of the overall decrease of PSI among all significant pairs in depressive patients with that in healthy subjects with a two-sample *t*-test. **(H)** Comparison of the average value of PSI decrease among all significant pairs in depressive patients with that in healthy subject with a two-sample *t*-test.

The number of electrode pairs exhibiting significantly greater PSIs during the target condition relative to that during the nontarget condition in the theta, alpha and beta frequency bands in the patients with depressive disorder group was larger than that in healthy controls. Conversely, the number of electrode pairs exhibiting significantly greater PSIs during the target condition relative to that during the nontarget condition in the delta frequency band in the patients with depressive disorder group was less than that in the healthy group (Figures 3A,B). The number of electrode pairs exhibiting significantly lower indices during the target condition relative to that during the nontarget condition in the delta and beta frequency band in the patients with depressive disorder group was greater than that in the healthy group. Conversely, the number of electrode pairs exhibiting significantly lower indices during the target condition relative to that during the nontarget condition in the theta and alpha frequency bands in the patients with depressive disorder group was smaller than that in the healthy group. (Figures 3E,F).

We also compared the PSI in the target condition with that in the nontarget condition from 1 to 20 Hz to investigate the difference of PSI modulation for all significant pairs between the groups. In the case that the participants showed significantly greater PSI in the target condition relative to the nontarget condition, the overall increase of PSI of all significant pairs (Figure 3C) in the patients with depressive disorder group was lower than that in the healthy group in the delta frequency band (e.g., at 1 Hz, *t*_(31)_ = 2.2675, *p* = 0.0359), but higher than that in the healthy group in the theta (e.g., at 6 Hz, *t*_(31)_ = 8.4631, *p* < 0.001), alpha (e.g., at 8 Hz, *t*_(31)_ = 4.7777, *p* < 0.001) and beta (e.g., at 14 Hz, *t*_(31)_ = 3.7720, *p* < 0.001) frequency bands. There was no significant group effect on the average value of increase in PSI of all significant pairs (Figure 3D). Moreover, in the case that the participants showed significantly lower PSI in the target condition relative to the nontarget condition, the overall decrease in PSI of all significant pairs (Figure 3G) in the patients with depressive disorders was higher than that in healthy controls in the delta (e.g., at 3 Hz, *t*_(31)_ = 3.7193, *p* < 0.001) frequency band, but lower in the theta (e.g., at 7 Hz, *t*_(31)_ = 5.0027, *p* < 0.001) and alpha (e.g., at 12 Hz, *t*_(31)_ = 3.6968, *p* < 0.001) frequency bands. There was no significant group effect on the average value for decrease in PSI among all significant pairs (Figure 3H).

### Topology of Delta Phase Synchronization Clusters

**Figure 4:**
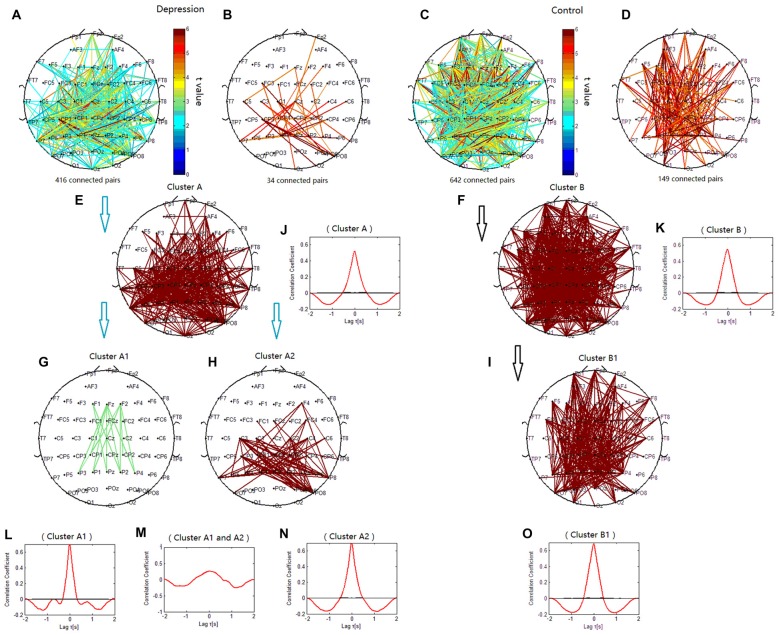
**Clustering of delta phase synchronization for both depressive patients and healthy controls based on the correlation coefficient of phase synchronization indices.** Lines represent significant PSI increase during target condition relative to that during nontarget condition (*P* < 0.05). **(A)** Task-dependent increase of the PSI at 1 Hz for depressive patients. **(B)** Electrode pairs with high significance of PSI increase (*t* > 4.5) in depressive patients. **(C)** Task-dependent PSI increase at 1 Hz for healthy controls. **(D)** Electrode pairs with high significance of PSI increase (*t* > 4.5) in healthy subjects. **(E)** Cluster A. **(F)** Cluster B. **(G)** Cluster A1. **(H)** Cluster A2. **(I)** Cluster B1. Drawing is the top view of the scalp. Each black spot signifies an electrode that was used for measurement. Panels **(J–O)** are the cross-correlation of phase synchronization within or between corresponding clusters.

The phase synchronization at 1 Hz showed a significant difference between the patients with depressive disorder group and the healthy group (*p* = 0.0359; Figures 4A,C). The lines represent significant increases of PSI during the target condition relative to that during the nontarget condition (*p* < 0.05). In the patients with depressive disorder group, the correlation coefficient of the PSIs of significant electrode pairs was tested for significance with a one-tailed one-sample *t*-test with *p* < 0.05. Delta phase synchronization could be classified into one independent cluster. This cluster A (Figure 4E) primarily linked 361 pairs of electrodes, including electrodes on the parietal, temporal and occipital sites and minor frontal sites. In the healthy group, delta phase synchronization could be classified into one independent cluster. This cluster B (Figure 4F) mainly linked the electrodes on the frontal and parietal, temporal, occipital sites and consisted of 611 pairs of electrodes. For both groups, the numbers of electrode pairs with a higher significance of PSI increases (*t* > 4.5) are shown (Figures 4B,D). The healthy group clearly showed more links between the posterior scalp sites and frontal sites than that in the patients with depressive disorder group.

When correlation coefficients were tested for significance with a one-tailed one-sample *t*-test (*p* < 0.01), cluster A in the patients with depressive disorder group was further classified into two independent clusters (cluster A1 and cluster A2). Cluster A1 (Figure 4G) mainly linked the electrodes between frontal and parietal sites at the midline and consisted of 14 pairs of electrodes. Cluster A2 (Figure 4H) consisted of 79 pairs of electrodes, which mainly linked the electrodes on the posterior parietal sites. In the healthy group, cluster B1 (Figure 4I) showed long-range phase synchronization between frontal sites and parietal/occipitoparietal sites and consisted of 175 pairs of electrodes. The phase synchronization network in the healthy group showed more functional connectivity between parietal sites and frontal sites than in the patients with depressive disorder group.

To verify the cross-correlation between time series of PSIs in the clusters, we computed the cross-correlation between cluster A1 and A2, and the cross-correlation of PSIs between the electrodes within each cluster. The cross-correlation coefficient between cluster A1 and cluster A2 was much smaller than that within cluster A1 and within cluster A2 (Figures 4L–N). The cross-correlation within clusters A, B and B1 were also showed (Figures 4J,K,O).

### Topology of Theta Phase Synchronization Clusters

**Figure 5:**
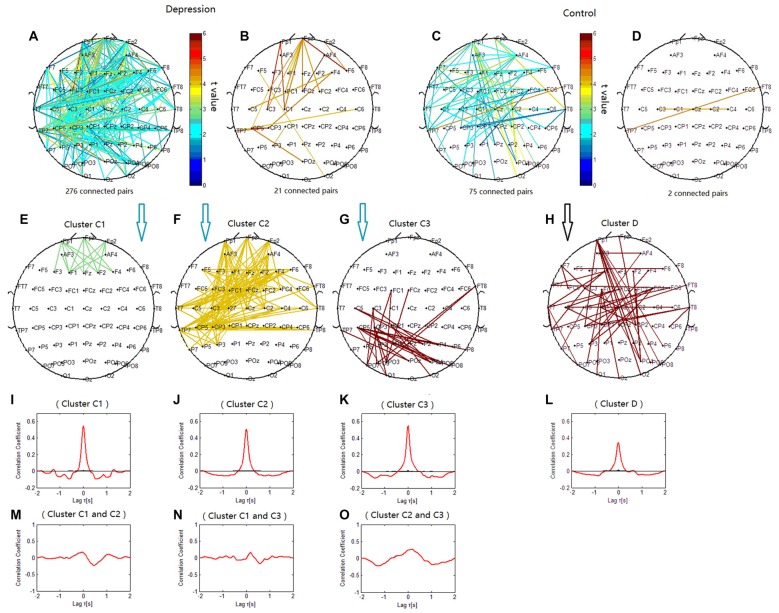
**Clustering of theta phase synchronization for both depressive patients and healthy controls.** Lines represent significant PSI increases during target condition relative to that during nontarget condition (*P* < 0.05). **(A)** Task-dependent PSI increase at 6 Hz for depressive patients. **(B)** Electrode pairs with high significance of PSI increase (*t* > 4.5) in depressive patients. **(C)** Task-dependent PSI increase at 6 Hz for healthy controls. **(D)** Electrode pairs with high significance of PSI increase (*t* > 4.5) in healthy subjects. **(E)** Cluster C1. **(F)** Cluster C2. **(G)** Cluster C3. **(H)** Cluster D. Panels **(I–O)** are the cross-correlation of phase synchronization within or between corresponding clusters.

The phase synchronization at 6 Hz was significantly different between the patients with depressive disorder group and the healthy group (*p* < 0.001). The lines in Figure 5 represent significant increases of PSI during the target condition relative to that during the nontarget condition (*p* < 0.05). There were more significant electrode pairs in the patients with depressive disorder group (276 pairs) than in the healthy group (75 pairs). When the correlation coefficient of the PSIs of significant electrode pairs was tested for significance with a one-tailed one-sample *t*-test (*p* < 0.05), the theta phase synchronization in the patients with depressive disorder group could be classified into three independent clusters (clusters C1, C2 and C3). Cluster C1 included 15 electrode pairs linked to prefrontal sites. Cluster C2 (Figure 5F) primarily linked the electrodes on the anterior brain regions including prefrontal sites, central sites, and electrodes on the left temporal sites and consisted of 131 pairs of electrodes. Cluster C3 included 26 electrode pairs and primarily linked electrodes on the left posterior brain regions. The theta phase synchronization in the healthy group could be classified into one independent cluster (cluster D). This cluster (Figure 5H) consisted of 53 pairs of electrodes indicating long-range phase synchronization between the anterior and posterior scalp sites and between the left and right hemispheres. Overall, these findings suggest that there was more anterior brain connectivity in the patients with depressive disorder group than in the healthy group.

**Figure 6:**
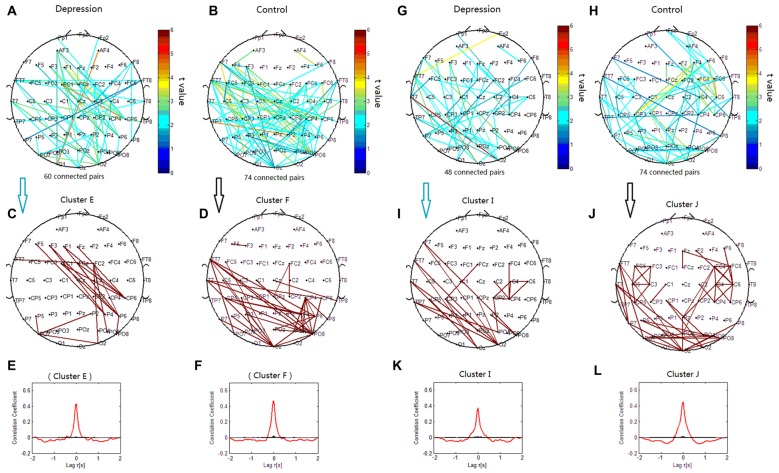
**Clustering of theta and alpha phase synchronization for both depressive patients and healthy controls in which the lines represent significant PSI decrease during target condition relative to that during nontarget condition (*P* < 0.05). (A)** Task-dependent PSI decrease at 7 Hz for depression disorders. **(B)** Task-dependent PSI decrease at 7 Hz for healthy controls. **(C)** Cluster E. **(D)** Cluster F. Panels **(E,F)** are the cross-correlation of phase synchronization within corresponding clusters. **(G)** Task-dependent PSI decrease at 12 Hz for depression disorders. **(H)** Task-dependent PSI decreases at 12 Hz for healthy controls. **(I)** Cluster I. **(J)** Cluster J. Panels **(K,L)** are the cross-correlation of phase synchronization within corresponding clusters.

The lines in Figures 6A–D represent significant decreases of PSI during the target condition relative to that during the nontarget condition at 7 Hz (*p* < 0.05) in both groups. When correlation coefficients were tested for significance with a one-tailed one-sample *t*-test (*p* < 0.05), the theta phase synchronization in the patients with depressive disorder group could be classified into one independent cluster (cluster E). Cluster E (Figures 6C,E) primarily linked the electrodes on the left frontal sites and those on the right parietal sites, and consisted of 18 pairs of electrodes. In the healthy group, the theta phase synchronization could be classified into one independent cluster (cluster F). Cluster F (Figures 6D,F) mainly linked the electrodes on left temporal sites and those on parietal or occipitoparietal sites, and consisted of 35 pairs of electrodes.

We verified that the cross-correlation between clusters C1 and C2 was obviously smaller than that within cluster C1 and within cluster C2. The cross-correlation between clusters C2 and C3 was smaller than that within clusters C2 and C3, and the cross-correlation between clusters C1 and C3 was smaller than that within cluster C1 and within cluster C3 (Figures 5I–L,N,O).

### Topology of Alpha Phase Synchronization Clusters

**Figure 7:**
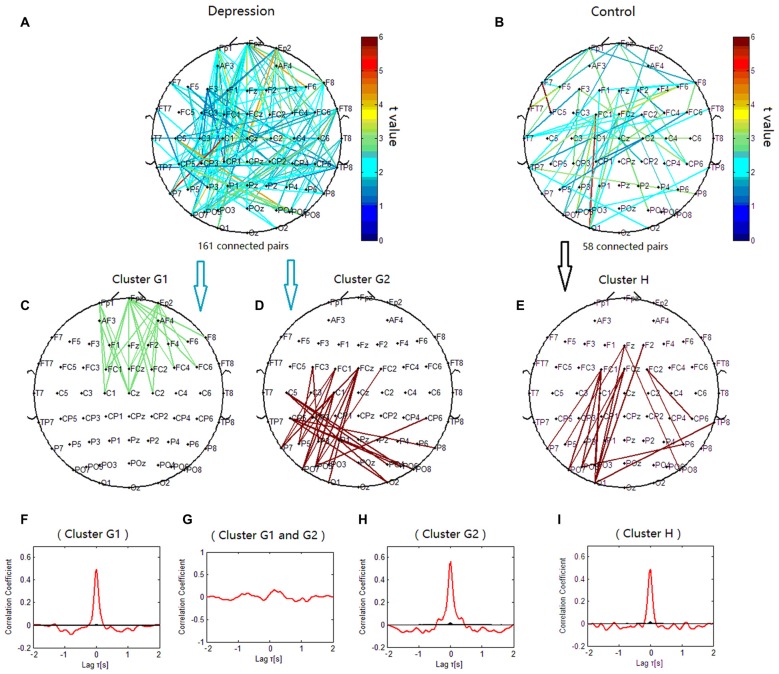
**Clustering of alpha phase synchronization for both depressive patients and healthy controls.** Lines represent significant PSI increase during target condition relative to that during nontarget condition (*P* < 0.05). **(A)** Task-dependent PSI increase at 8 Hz for depression disorders. **(B)** Task-dependent PSI increase at 8 Hz for healthy controls. **(C)** Cluster G1. **(D)** Cluster G2. **(E)** Cluster H. Panels **(F–I)** are the cross-correlation of phase synchronization within or between corresponding clusters.

The phase synchronization at 8 Hz was significantly different between the patients with depressive disorder group and the healthy group (*p* < 0.001; Figures 7A,B). The lines of Figure 7 represent significant increases of PSI during the target condition relative to during the nontarget condition (*p* < 0.05) at 8 Hz in both groups. When the correlation coefficient of the PSIs for significant electrode pairs were tested for significance with a one-tailed one-sample *t*-test (*p* < 0.05), the alpha phase synchronization in the patients with depressive disorder group could be classified into two independent clusters (cluster G1 and cluster G2). Cluster G1 (Figure 7C) mainly linked the electrodes on the prefrontal sites including the frontopolar, frontal and central sites, particularly in the right hemisphere, and consisted of 24 pairs of electrodes. Cluster G2 (Figure 7D) mainly linked the electrodes on the posterior scalp sites between frontocentral/central sites and parietal/parieto-occipital sites, and consisted of 24 pairs of electrodes. However, in the healthy controls, the theta phase synchronization could be classified into one independent cluster (cluster H). This cluster (Figure 7E) consisted of 16 pairs of electrodes, primarily linking the electrodes on the posterior scalp sites between frontocentral sites and parietal/parieto-occipital sites. These results suggest that phase synchronization connectivity was more prevalent in prefrontal sites in the patients with depressive disorder group than in the healthy group.

We also observed significant decrease of PSI during the target condition relative to during the nontarget condition for phase synchronization at 12 Hz (*p* < 0.05; Figures 6G–J). When correlation coefficients were tested for significance with a one-tailed one-sample *t*-test (*p* < 0.05), the alpha phase synchronization in patients with depressive disorder could be classified into one independent cluster (cluster I). Cluster I (Figures 6I,K) primarily linked the electrodes on the left frontal/temporal and right parieto-occipital or occipital sites, and consisted of 22 pairs of electrodes. However, the theta phase synchronization in healthy controls could be classified into one independent cluster (cluster J). Cluster J (Figures 6J,L) primarily consisted of 49 pairs of electrodes, and was similar to cluster I, except that there were more links around the right central scalp region between the right frontal sites and right centroparietal sites in cluster J. There was a smaller decrease of PSI between the right frontal and centroparietal sites in the patients with depressive disorder group than in the healthy group.

### Topology of Beta Phase Synchronization Clusters

**Figure 8:**
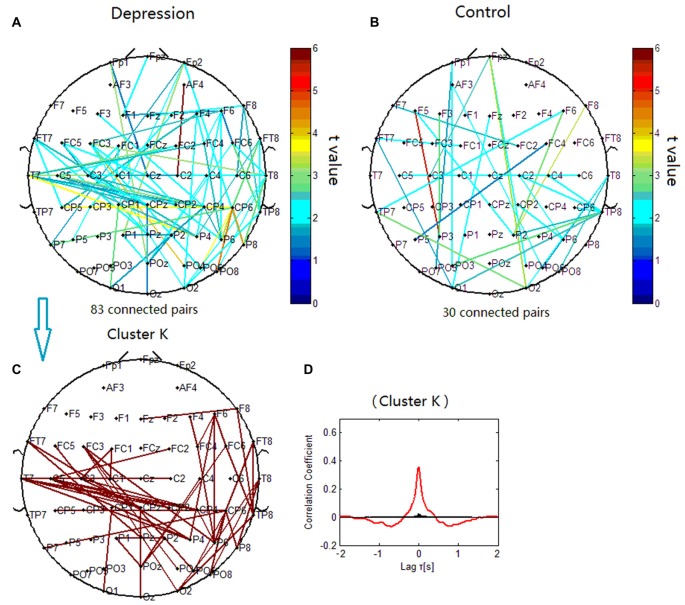
**Clustering of beta phase synchronization for both depressive patients and healthy controls.** Lines represent significant PSI increase during target condition relative to that during nontarget condition (*P* < 0.05). **(A)** Task-dependent PSI increase at 14 Hz for depression. **(B)** Task-dependent increases of the EEG PSI at 14 Hz for healthy controls. **(C)** Cluster K. Panel **(D)** is the cross-correlation of phase synchronization within cluster K.

The phase synchronization at 14 Hz was significantly different between the patients with depressive disorder group and the healthy group (*p* < 0.001; Figures 8A,B). When correlation coefficients were tested for significance with a one-tailed one-sample *t*-test (*p* < 0.05), beta phase synchronization in the patients with depressive disorder group could be classified into one independent cluster (cluster K). Cluster K (Figures 8C,D) consisted of 55 pairs of electrodes, and primarily linked the electrodes on the frontocentral sites, central sites and centroparietal sites on the left hemisphere, and also included several long-range connections between the right frontal and right parietal sites. The beta phase synchronization in the healthy group was unable to be classified into one independent cluster. All 30 significant electrode pairs mainly included several long-range connections between the anterior scalp sites and posterior scalp sites. We found increased frontal connectivity in the right hemisphere and increased connectivity around the central scalp region on the left hemisphere in the patients with depressive disorder group.

## Discussion

We found that in the visual oddball paradigm, patients with depressive disorder demonstrated higher theta, alpha and beta ERPCOH in the frontal/prefrontal regions compared with healthy controls. These findings reflect a strengthened functional connectivity within prefrontal/frontal regions and between prefrontal/frontal and temporal or parietal regions in the frequency bands associated with major depression. There was also a decreased functional connectivity between the frontal and parietal/temporal/occipital sites in the delta frequency band in the patients with depression. Further, we supposed that there may exist a compensatory mechanism in patients with depression, their functional connectivity in high-frequency bands (theta, alpha and beta) was increased to compensate for functional deficits of a deficient low-frequency connection between the frontal and parietal/temporal/occipital regions to maintain normal cognitive performance.

### Task-Dependent Functional Connectivity for Patients with Depression

A smaller target-dependent PSI increase in delta band, and a larger target-dependent PSI increase in theta, alpha and beta bands were found in patients with depressive disorders than in healthy participants, as well as, a smaller target-dependent PSI decrease in the theta and alpha bands was also found in patients with depressive disorder than in healthy participants.

A number of studies of resting-state quantitative EEG have reported increased neurophysiologic connectivity associated with depression, including higher theta and alpha coherence in connections between frontopolar and temporal or parieto-occipital regions, and higher beta coherence primarily in connections within and between electrodes overlying the dorsolateral prefrontal cortical or temporal regions (Leuchter et al., 2012). A advanced measure of EEG signal synchronization for the “resting state” indicated that major depressive disorder shows widespread and significant increases in theta and alpha band inter-area functional connectivity (Fingelkurts et al., 2007). These data provide specific evidence of higher high-frequency band coherence, consistent with our finding about the increase of ERPCOH in theta, alpha and beta bands in patients with depression.

Previous comparative analysis demonstrated that patients with depression had fewer global delta oscillations than those without depression (Fingelkurts and Fingelkurts, 2015). In the present oddball paradigm, we also found a smaller event-related PSI increase in the delta band in major depression.

### Topological Aspects of Task-dependent Functional Connectivity in Patients with Depression

Previous studies on cognitive dynamics indicated that the oscillatory responses of P300 are composed of delta, theta and alpha responses primarily for the target stimuli (Oniz and Basar, 2009), while emphasized the role of circuits between frontal, parietal and temporal areas for the generation of P300. P3a and P3b indicate a circuit pathway between the frontal and temporal/parietal brain areas (Soltani and Knight, 2000; Polich, 2007). P300 amplitude is affected by temporal-parietal junction integrity, as its absence greatly reduces component size over the parietal area (Li et al., 2010, 2015; Bruder et al., 2012). Further, a reduction in P300 amplitude in patients with depression may indicate that the circuit pathway between the frontal and parietal areas is abnormal (Kemp et al., 2010).

In the present study, compared with control subjects, patients with depression showed decreased P3b amplitude at parietal site. (Figure 1A). The decrease of P300 subcomponent in delta band was also observed at the parietal site in the patients (Figure 1D). Moreover, cluster A (Figure 5) indicates insufficient functional connectivity between the frontal and parietal/temporal/occipital sites in patients with depressive disorders in the delta frequency band. Taking all of the discussed information in this subsection together, we supposed that the reduction of P3b at the parietal site in patients with depression during this oddball paradigm may arise from the decreased low-frequency (delta band) functional connectivity between the frontal and parietal/temporal/occipital sites, and this reduction of connection may reflect functional deficit during the top-down attentional process.

Compared with healthy subjects, patients with depressive disorders also showed increased P3b amplitude at the right frontal site (Figure 1A). Likewise, the increase of P300 subcomponent in theta, alpha and beta bands was observed at the frontal site in the patients (Figure 1D). Further, patients with depression showed excessive functional connectivity within the prefrontal/frontal regions and between the frontal and temporal regions in the theta band (clusters C1 and C2), within the prefrontal/frontal regions in alpha band (cluster G1), and within the frontal and central regions in beta band (cluster K). Taking together, we supposed that the increased P300 amplitude at the right frontal site was due to excessive frontal/prefrontal functional connectivity in the theta, alpha and beta frequency bands. This finding is consistent with the increased index of structural synchrony in the right frontal regions in the theta and alpha bands, as well as hyperactivity in the right anterior cortical regions (Fingelkurts et al., 2007). We interpreted the increase of above connections as the following deterioration in major depression. While the ability to modulate alpha activity has been linked to the ability to shift and focus attention and meet executive demands (Sauseng et al., 2005; Uhlhaas and Singer, 2006; Del Percio et al., 2011), the increased frontal alpha connection in the present results may reflect dysfunction in top-down control circuit that is mediated by alpha activity (Leuchter et al., 2012). Modulation of beta activity has been associated with response preparation and cognitive control (Engel and Fries, 2010), an excessive beta connection in this study may reflect impaired cognitive control in major depression (Leuchter et al., 2012). The anterior cingulate cortex (ACC) is involved in conflict monitoring and error detection that evokes theta activity (Pizzagalli, 2011), increased theta connection in central scalp sites may reflect disruption of functional connectivity in frontal-cingulate pathways in major depression (Pizzagalli et al., 2003; Fingelkurts and Fingelkurts, 2015).

This finding of increased frontal/prefrontal activities is consistent with previous studies showing enhanced frontal and ACC activities in patients with depression during challenging cognitive tasks, such as the n-back task (Harvey et al., 2005; Rose et al., 2006). The excessive activation of the frontal and the ACC associated with normal performance in depressed subjects in those studies was interpreted as an excess of cognitive control or subject’s task engagement, more brain activation is needed in the processing regions in major depression to get normal functioning during these tasks.

Similar with above-mentioned studies, the patients with depression in the present study did not demonstrate deteriorating performance. The increased frontal connection in major depression during this attentional task may also reflect an effort to compensate for deficient functional connectivity. Therefore, we supposed a compensatory mechanism for functional deficit in patients to maintain normal cognitive performance. The patients increased their high-frequency EEG activities (theta, alpha and beta bands) in the frontal regions and connection between the frontal and posterior regions. These changes could compensate for their impairment of low-frequency (delta band) functional connectivity between the frontal and parietal/temporal/occipital regions during attentional process. Although some researchers also found increased frontal activity during cognitive tasks in previous studies (Harvey et al., 2005; Rose et al., 2006; Steele et al., 2007; Leuchter et al., 2012), the present EEG study decomposed the whole disorganized brain activity into the low-frequency oscillation reflecting cognitive deficit and the high-frequency oscillations which may reflect compensatory efforts. Therefore, we could expand the knowledge about these features of cognitive processes in major depression. It implied that the alteration of reorganization of the EEG oscillation could contribute to the evaluation of the effectiveness of the treatment or therapeutic strategy for depression. A treatment modality for major depression could be evaluated effective if the low-frequency hypoactivity and the high-frequency hyperactivity of EEG oscillation during the attentional task were remitted after the treatment, this supposition is required to be tested by a follow-up study in the future.

Additionally, in cluster D in healthy participants, the left frontal sites showed increased PSI activity compared with the right frontal sites, which was not seen in cluster C1 and C2 in the patients with depression. When compared with cluster F in healthy participants, cluster E in the patients with depressive disorder also showed significant PSI decreases in the left frontal sites. These results reflect the fact that there were no signs of increased PSI in the left frontal regions in the patients with depression, which were observed in healthy participants (Davidson et al., 2000; Coan and Allen, 2003; Davidson, 2004). This finding is consistent with the approach-withdrawal theoretical framework, which hypothesizes that the left frontal lobe regions are hypoactive in depression, implying potential system controls of behavioral motivation (Davidson, 1998; Fingelkurts and Fingelkurts, 2015).

## Conclusion

In patients with depressive disorder, we found evidence of: (a) decreased PSI in low-frequency (delta frequency band) activity indicates impairment of the connection between the frontal and parietal/temporal/occipital regions; and (b) increased frontal and prefrontal PSI of the theta, alpha and beta band activities that may operate in compensation for deficit of functional connectivity during the delta synchronization process. We supposed a compensatory mechanism for functional deficit to maintain normal cognitive performance in major depression, the high-frequency EEG oscillations in the frontal region and high-frequency connection between the frontal and posterior regions were increased to compensate for their impairment of low-frequency connection between the frontal and parietal/temporal/occipital regions. These findings may provide a potentially useful indication to evaluate the effectiveness of therapies for depression.

## Author Contributions

LY and YH designed this study. LY, WW, CK and YZ conducted this experiment. LY, CK and XQ prepared all tables and figures. LY, YH and CK wrote the main manuscript text. All authors reviewed the manuscript.

## Conflict of Interest Statement

The authors declare that the research was conducted in the absence of any commercial or financial relationships that could be construed as a potential conflict of interest.

## Footnotes

^1^http://sccn.ucsd.edu/wiki/Chapter_11:_Time/Frequency_decomposition
